# Supplementary material for: Identification of Freezing-Responsive microRNAs and Their Targets in Chinese Jujube by Small RNA and Degradome Sequencing
Source: Int J Mol Sci. 2026 Sep 4;27(17):7912. doi: 10.3390/ijms27177912 (PMC13566890; doi:10.3390/ijms27177912)
Supplement: Supplementary file 1 [file ijms-27-07912-s001.zip › Supplementary Material S2/novel_miR_175.pdf]

Provisional ID	:	novel_miR_175
Score total	:	1.8
Score for star read(s)	:	-1.3
Score for read counts	:	0
Score for mfe	:	1.5
Score for randfold	:	1.6
Score for cons. seed	:	
Total read count	:	73
Mature read count	:	64
Loop read count	:	9
Star read count	:	0

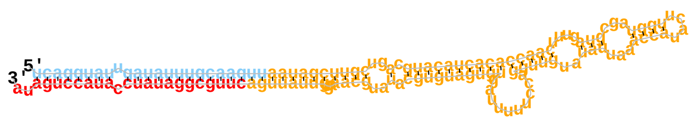

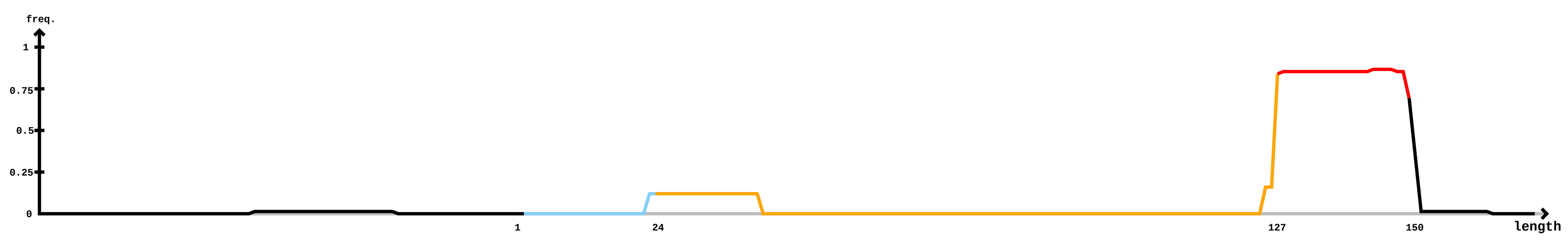

Star

Mature

```
5'- ugccaaaaauccaugguuguuucuaguuugaaaaaggcauguuuaggaguuuagaauacauauacauguacauccccuaucagguauugauauuugcaaguuaauagcuugcugacguacaucacaccaacuuugaugcgaugguucauaccaaauuauauguuggaccuuuuuagugugaugugcauaugcaaaguguuauugacuugcggauauccauaccugauaauuauuuuguauauauuucugc -3'   exp
    ..((.(((...((((.((.((((......)))).)).))))))).))....(((((...(((((((..........(((((((((.((((((((((((((((((((((((..(.((((((((((((((....(((...((((....))))...)))..)))))..........))))))))).)..))))...))))))))))))))))))))).))))))))))......)))))))..))))).   reads  mm  sample
    ..................................aggcauguuuaggaguuuagaaua................................................................................................................................................................................................   1  0  LT-3
    ....................................................................................................uuaauagcuugcugacgua..................................................................................................................................   9  0  LCK-3
    ...........................................................................................................................................................................................................gacuugcggauauccauaccugau.......................   2  0  JCK-1
    ...........................................................................................................................................................................................................gacuugcggauauccauaccugau.......................   7  0  JCK-2
    ...........................................................................................................................................................................................................gacuugcggauauccauaccugau.......................   2  0  JCK-3
    ...........................................................................................................................................................................................................gacuugcggauauccauaccuNau.......................   1  1  JCK-1
    .............................................................................................................................................................................................................cuugcggauauccauaccugaua......................   24  0  LCK-2
    .............................................................................................................................................................................................................cuugcggauauccauaccugaua......................   22  0  LCK-1
    .............................................................................................................................................................................................................cuugcggauauccauaccugaua......................   5  0  LT-2
    ..............................................................................................................................................................................................................uugcggauauccauaccug.........................   1  0  JCK-1
    .............................................................................................................................................................................................................................ccugauaauuauuuuguaua.........   1  0  LT-2
```
